# Antioxidant nanoparticles on osteoarthritis and rheumatoid arthritis: a systematic review

**DOI:** 10.1186/s12951-026-04876-3

**Published:** 2026-08-17

**Authors:** Naila Qamar, Lucia Baixauli Marin, Krista Juurikka, Hanna Karjalainen, Janne T.A. Mäkelä, Antoine Dufour, Mikko A.J. Finnilä, Pirjo Åström

**Affiliations:** 1 Research Unit of Health Science and Technology, Faculty of Medicine, University of Oulu, Oulu, FI-90014 Finland; 2 Research Unit of Biomedicine and Internal Medicine, Faculty of Medicine, University of Oulu, Oulu, FI-90014 Finland; 3 Faculty of Biochemistry and Molecular Medicine, University of Oulu, Oulu, FI-90014 Finland; 4 Department of Technical Physics, University of Eastern Finland, Kuopio, FI-70211 Finland; 5 Departments of Physiology and Pharmacology & Biochemistry and Molecular Biology, Cumming School of Medicine, University of Calgary, Calgary, AB T2N 4N1 Canada; 6 Medical Research Center, Oulu University Hospital and University of Oulu, Oulu, Finland; 7 Biocenter Oulu, University of Oulu, Oulu, FI-90014 Finland; 8 Infotech Oulu, University of Oulu, Oulu, FI-90014 Finland

**Keywords:** Antioxidant, Nanoparticles, Rheumatoid arthritis, Osteoarthritis, Therapeutics

## Abstract

Nanoparticle (NP)-based therapies, including hydrogel, polymeric, and inorganic forms (carbon, metal, and metal oxide NPs/frameworks), have attracted significant interest for biomedical applications due to their antioxidant and enzyme-mimetic activities. This systematic review aims to identify different antioxidant NPs used in preclinical models of rheumatoid arthritis (RA) and osteoarthritis (OA) and summarize their biosafety, anti-inflammatory, anti-arthritic, and antioxidant effects. Studies published up to December 2024 were systematically searched across PubMed, Cochrane Library, Web of Science, Scopus, the WHO International Clinical Trials Platform, and Embase. The study quality was assessed using SYRCLE’s risk of bias tool for animal studies and custom quality assessment criteria for in vitro studies. Altogether, 516 studies were retrieved, and 26 met the inclusion criteria and were incorporated into the review. Studies showed that selenium, silver, and gold are frequently used NPs for RA, while cerium, iron, and manganese oxide NPs are commonly employed for OA. Generally, NPs demonstrate the potential to increase antioxidant activity and reduce inflammation and the structural degeneration of joints without evident short-term systemic toxicity in both conditions. In summary, pre-clinical evidence suggests that NP-based therapies might offer a promising approach to managing arthritis by targeting oxidative stress and inflammation. However, their long-term safety requires further evaluation.

**Graphical abstract:**

**Figure:**
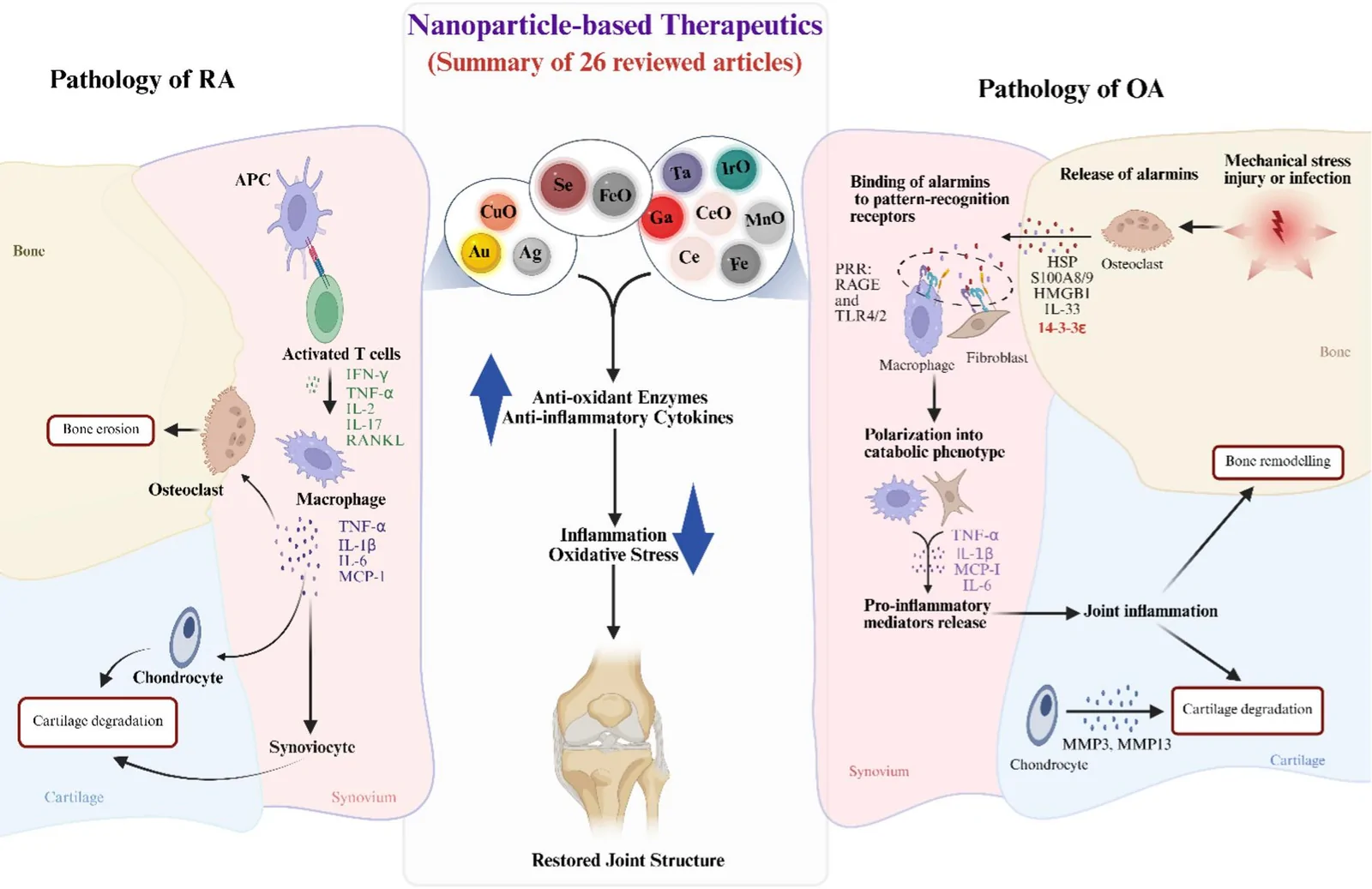

**Supplementary Information:**

The online version contains supplementary material available at https://doi.org/10.1186/s12951-026-04876-3.

## Introduction

Osteoarthritis (OA) and rheumatoid arthritis (RA) are not just degenerative or autoimmune joint diseases but involve redox-driven components, where persistent oxidative stress drives synovial inflammation and cartilage degradation [1, 2]. Multiple factors contribute to the pathogenesis of OA, including aging, genetic predisposition, obesity, biomechanical disturbances (trauma), and environmental factors. These factors interact with cellular and molecular pathways that drive joint damage through various tissue- and cell-derived mediators [3–5]. In OA, excessive reactive oxygen species (ROS) lead to enhanced apoptosis of chondrocytes, disrupt insulin receptor substrate 1 (IRS-1) / phosphatidylinositol 3-kinase (PI3K) / AKT serine/threonine kinase (AKT) signaling, and impair matrix synthesis. In RA, nicotinamide adenine dinucleotide phosphate (NADPH)-derived ROS strengthens (MAPK), nuclear factor kappa-light-chain-enhancer of activated B cells (NF-κB), and janus kinase (JAK) - signal transducer and activator of transcription (STAT) signaling and sustains immune cell recruitment and cytokine production [1, 2].

OA is not restricted solely to the articular cartilage and subchondral bone but may also affect the adjacent connective tissue and synovium, leading to damaged joint structures and synovitis [3–6]. OA progresses slowly and ultimately leads to joint pain, disability, and, in some cases, immobility [4]. RA is an autoimmune disease characterized by swelling, stiffness, and pain (inflammation) in tissues. Subsequent damage to bone and cartilage in synovial joints leads to impairment and disability [7, 8]. The exact cause of RA is still unclear, but the environmental and genetic factors are suspected to trigger immunomodulation inappropriately and initiate the synovial inflammatory cascade, resulting in joint damage [7, 8]. OA and RA are common arthritic diseases with a significant impact on healthcare systems around the world [3–5]. The prevalence of OA is anticipated to reach approximately 25% in the older population by 2030 [6]. RA affects women more often than men (2.5:1), with a 1% incidence worldwide. It can occur at any age but is more prevalent in the 40–70 age range [8].

Current therapies (e.g., disease-modifying antirheumatic drugs) are generally used to treat RA, while analgesics and non-steroidal anti-inflammatory drugs are commonly prescribed for RA and OA. No effective curative therapies are available for arthritis; at best, current treatments offer limited protection against disease progression [3, 4, 6, 7] and may cause adverse effects such as renal dysfunction and liver toxicity with long-term use [7, 9]. Free radicals and their derivatives, generated through metabolic reactions in synoviocytes and chondrocytes, can accumulate in the synovial joint and cause inflammation, structural damage, and cell death [10].

ROS are the chemically reactive oxygen-containing molecules characterized by unpaired electrons, including free radicals such as singlet oxygen (¹O₂), superoxide anion radical (•O₂⁻), hydroxyl radical (•OH), and non-radical oxidants like hypochlorous acid (HOCl) and hydrogen peroxide (H₂O₂) [11]. ROS are also produced in the cells at low levels to maintain their homeostasis and essential functions [12]. The balance in the ROS level is maintained by the enzymes of the cellular antioxidant defense system (endogenous and exogenous antioxidants) such as vitamin C, vitamin E [11], glutathione peroxidase (GPx), superoxide dismutase (SOD), catalase (CAT) [12–14], peroxiredoxins (Prxs), and NADPH ubiquinone oxidoreductase (NQO1) [12]. An imbalance in this mechanism can lead to increased production of chemokines and inflammatory cytokines, thereby driving oxidative stress. Subsequent oxidative damage to cellular macromolecules leads to apoptosis of chondrocytes—a major contributor to the progression of OA [5, 12].

Chondrocytes have limited self-renewal and are naturally quiescent [12, 15]. Upon damage, they secrete a plethora of proinflammatory chemokines and cytokines, which increase the expression of aggrecanases and collagenases, leading to the degradation of cartilage ECM [12]. Lower activity of the glutathione antioxidant system increases chondrocytes’ susceptibility to oxidative stress-induced cell death through apoptosis mediated by the PI3K/AKT and c-jun N-terminal kinase (JNK) signaling pathways. The ROS and nitric oxide (NO) regulate this pathway. NO increases the production of various proinflammatory cytokines (interleukin (IL) −1 and tumor necrosis factor (TNF)-α), leading to the activation of the NF-κB pathway, which is involved in the inflammatory response of the synovial lining and vascular endothelium, a characteristic feature of synovium in OA and RA patients [12, 16]. Such oxidative damage due to ROS imbalance is commonly associated with OA and RA, diabetes, neurodegeneration, cardiovascular disease, and cancer [12, 14].

Antioxidants are natural reducing agents that inhibit ROS formation and eliminate the free radicals and their derivatives [10]. Therefore, antioxidant-based therapies have emerged as a promising approach in managing ROS-related inflammation. Over recent decades, a variety of natural and synthetic antioxidant compounds have been identified [11]. Antioxidant vitamins (e.g., vitamin E, vitamin C [ascorbic acid], plant polyphenols, and thiols) can reduce the oxidative stress-related arthritis progression by neutralizing the ROS in joints [17]. To target oxidative stress in OA, antioxidant supplements, free radical scavengers, and mediators of various oxidative stress pathways are being studied for OA treatment with varying success. Despite a strong pre-clinical rationale, conventional antioxidant therapies have shown limited clinical efficacy, mainly due to suboptimal bioavailability, rapid clearance, and inadequate tissue specificity, prompting interest in nanotechnology-based antioxidant delivery systems [6].

Nanotechnology is a growing field in which engineering and the natural sciences are combined to design, synthesize, characterize, and apply the devices and materials from technology to medicine with molecular precision [18, 19]. Nanotechnology focuses on designing new materials with distinct structural and compositional properties through atomic/molecular rearrangements at 1–100 nm scale [20, 21] including nanoparticles (NPs) (metallic [Mt], metal oxide [MO], and polymeric), quantum dots, dendrimers, liposomes, and carbon-based materials (graphene and carbon nanotubes) [22].

The unique physicochemical properties of NPs enable them to incorporate multiple functionalities (e.g., drug payloads, imaging agents, or targeting ligands), allowing simultaneous delivery, tracking, and targeting [23] and developing into new branches (e.g., theranostics and nanomedicine) with the potential to replace current technologies [18]. Furthermore, it shows promising potential for developing vaccines, therapeutics, and diagnostic strategies, as its biomedical applications demonstrate, including delivery systems, in situ and multimodal imaging, and immune system regulation [19]. For instance, the non-viral NPs platform, especially lipid-based NPs and cationic liposomes, has been used safely and efficiently to deliver mRNA vaccines [24].

In recent years, various NP-based systems have been investigated for their ability to modulate the arthritic microenvironment, such as inorganic NPs, nanocomposite hydrogels, and biodegradable polymeric NPs [25]. Inorganic NPs (e.g., metal-based, carbon-based, and metal-organic frameworks [MOFs]) have attracted significant attention due to their intrinsic enzyme-mimicking activities and efficient ROS-scavenging properties in biomedical applications [11]. Currently, the substances most encapsulated in NPs by researchers fall into three main categories: metal oxides, non-metals, and biologically active compounds [26].

From this perspective, Mt- and MO-NPs represent a fundamentally different therapeutic concept. Apart from their known applications in imaging, catalysis, drug delivery, and sensing, various Mt-NPs (gold [Au], silver [Ag], selenium [Se], platinum [Pt], ruthenium [Ru], and palladium [Pd]) and MO-NPs (iron oxide [Fe_3_O_4_], cerium oxide [CeO_2_], titanium oxide [TiO_2_], manganese dioxide [MnO_2_], copper oxide [CuO], and zinc oxide [ZnO]) exhibit intrinsic redox activities resulting from their surface modifications, various oxidation states, and high surface-to-volume ratios. The catalytically active nanozyme behavior of NPs differs completely from that of conventional nanocarriers, which are designed mainly to transport drugs. MnO_2_ NPs possess microenvironment-reactive catalytic activity; CeO_2_NPs undergo reversible redox cycling (Ce^3+^/Ce^4+^), while Se-, Pd-, and Pt-NPs exhibit the CAT-, GPx-, and SOD-like properties [2, 27]. These NPs can suppress ROS-mediated NF-κB activation, regulate the inflammatory cytokine cascade, and attenuate MAPK phosphorylation by modulating redox balance through scavenging H₂O₂ and superoxide [2].

Notably, Mt- and MO-NPs possess dual pro- and antioxidant properties depending on size, composition, surface chemistry, oxidation state, and dose [27, 28]. Smaller NPs usually exhibit improved ROS modulation and enhanced catalytic surface reactivity, while the excessive ROS modulation can induce oxidative stress. Therefore, nanoengineering (e.g., size optimization and surface chemistry) is essential to achieve therapeutic antioxidant activity while minimizing toxicity. Though previous reviews have broadly addressed ROS-modulating NP-induced oxidative stress [1, 28], a systematic synthesis of arthritis-specific intrinsic antioxidant Mt- and MO-NPs remains lacking. In particular, the mechanistic junction between OA/RA-specific inflammatory signaling pathways and NPs-mediated redox homeostasis has not been critically integrated.

This review addresses this gap by focusing on antioxidant Mt- and MO-NPs in RA and OA; by mechanistically linking NPs-mediated ROS modulation to MAPK, PI3K/Akt, and NF-κB signaling in joint tissues; and by critically assessing their surface, size, and oxidation-dependent biological effects.

## Methodology

### Protocol and registration

The Cochrane Handbook for Systematic Reviews of Interventions [29] and the PRISMA (Preferred Reporting Items for Systematic Review and Meta-Analyses) guidelines were followed in this systematic review [30]. The study protocol was registered in PROSPERO (CRD420250485983).

### Search strategy

The databases of Cochrane Library, PubMed, Scopus, Web of Science, the WHO International Clinical Trials Platform, and Embase were searched for studies published up to December 2024. The full search strings utilized Boolean operators to combine terms related to the disease state and the intervention. For example, the primary search string across PubMed and Web of Science was: (“osteoarthritis” OR “rheumatoid arthritis” OR “arthritis”) AND (“antioxidant nanoparticles” OR “nanoparticles” OR “nanozymes”) AND (“therapy” OR “treatment”). Database-specific syntax adaptations (such as MeSH terms in PubMed and Emtree terms in Embase) were applied to ensure comprehensive retrieval. The complete, detailed search strategy for each database is provided in Table S1. After the initial search, two reviewers (NQ & HK) independently conducted further screening according to the inclusion and exclusion criteria.

### Inclusion and exclusion criteria

The studies that met the following criteria were included: (1) In vitro or in vivo arthritis models; (2) NPs as intervention; (3) Control drug; (4) Reported antioxidant enzyme activity, ROS / reactive nitrogen species (RNS) levels, histology and/or joint swelling; (5) Published in English; (6) Original research; (7) Published up to December 2024. The excluded studies met the following criteria: (1) In silico studies; (2) Reviews, editorials, notes, duplicates; (3) Non-antioxidant Mt-/MO-NPs used only for drug delivery; (4) Non-NP materials; (5) No control drug in RA studies.

Retrieved articles were imported into Covidence [31] for systematic screening based on predefined criteria. Initially, articles were screened based on their titles and abstracts, and irrelevant studies were excluded (NQ & HK). Subsequently, two reviewers (NQ & HK) independently evaluated the full texts against the inclusion and exclusion criteria, and two reviewers (MF & PÅ) resolved conflicts arising from title/abstract screening via consensus. The articles meeting the eligibility criteria were included for data extraction (by NQ, HK, & LBM), followed by comprehensive analysis and discussion.

### Quality assessment

Three reviewers (NQ, HK & LBM) independently assessed the quality of each study using SYRCLE’s risk of bias (RoB) tool (Animal studies) [32] and Custom Quality Assessment Criteria (in vitro studies). A consensus-oriented final decision was made in case of any conflict. The quality of in vivo studies was evaluated according to sequence generation, baseline characteristics, and allocation concealment (selection bias); random housing and blinding (performance bias); blinding and random outcome assessment (detection bias); incomplete outcome data (attrition bias); selective outcome reporting (reporting bias); and other sources of bias.

For in vitro studies, a custom quality assessment checklist comprising nine questions was utilized. Each item was assigned nine points based on cell characterization and randomization (selection bias); blinding and replication (performance bias); blinding and dose-response (detection bias); statistical analysis; incomplete outcome data; and selective outcome reporting (attrition & reporting bias).

The studies were assessed based on responses to the abovementioned questions: “high” indicated a high risk of bias; “low” indicated a low risk of bias; and “unclear” indicated an unclear risk of bias. To present the RoB findings, the Robvis online visualization tool [33] was used to create a summary and traffic light plots to clearly illustrate bias risk across the included studies.

### Data extraction

Three reviewers (NQ, LBM, & HK) independently extracted data on the NPs’ synthesis, formulation, administration, in vitro and in vivo antioxidant effects, and biodistribution analysis. Two reviewers (MF & PÅ) arbitrated any disagreements.

### Data synthesis

Quantitative meta-analysis was infeasible due to substantial heterogeneity in NP size, composition, surface functionalization, formulations, concentrations/dosing regimens, administration routes, treatment duration, disease models, and measured outcomes. Therefore, a narrative synthesis approach was adopted to compare and summarize the findings across the included studies.

### Data visualization

Alluvial and scatter plots were generated using the R statistical language (version 4.4.0 [34]; with data table (version 1.17.0); dplyr (version 1.1.4); ggalluvial (version 0.12.5); and ggplot2 (version 4.0.0) packages. GraphPad Prism version 10 (GraphPad Software, San Diego, CA, USA) was used to generate graphs of the yearly distribution of included studies.

## Results and discussion

### Study selection

**Fig. 1:**
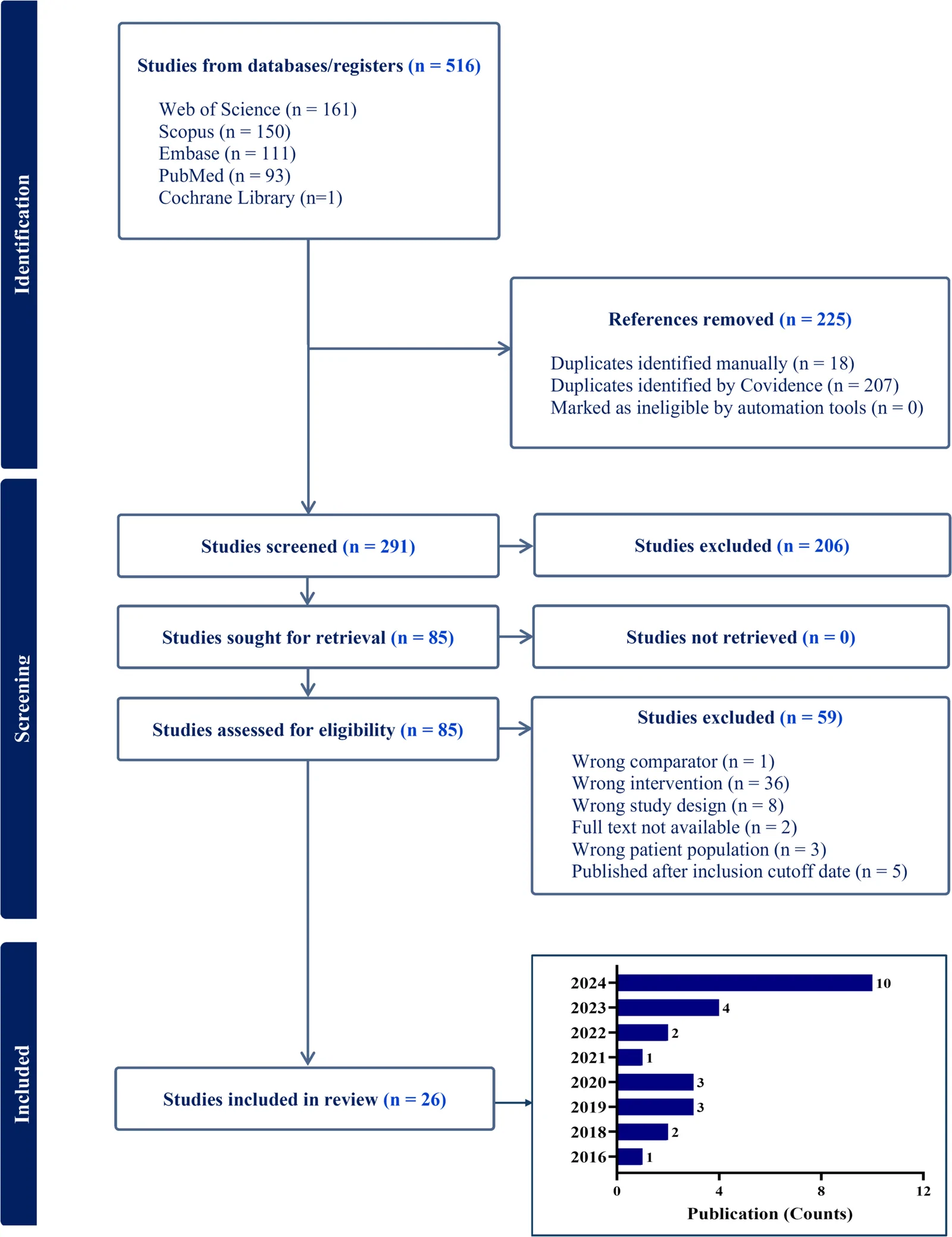
PRISMA flowchart of study selection from Covidence

From the initial literature search, 516 records were imported into Covidence for screening. Among these, 225 duplicate records were removed. The remaining 291 records proceeded to the title and abstract screening phase, during which 206 were excluded based on predefined inclusion and exclusion criteria. The remaining 85 studies underwent full-text screening: 26 [35–60] met the criteria and were included in data extraction. The included studies were published from 2016 to 2024; of these, 12 focused on RA [38, 42–47, 52, 54, 57, 59, 60] and 14 on OA [35–37, 39–41, 48–51, 53, 55, 56, 58]. Figure 1 illustrates the flowchart of the process and Table 1 summarizes all the included studies.

**Table 1:** Summary of the main information extracted from the included studies

Disease	Model		Nanoparticles		Study group			Route	Duration (days)		Outcomes		Ref.
Pre-Clinical Studies (included studies)													
RA	Balb/c		SeNPs		SeNP (2,5, 5, 10, 20 mg/kg), healthy, arthritis, drug control (Lef)			Oral	14		Antioxidant enzymes, joint inflammation, biochemical index		[60]
RA	Balb/c		SeNPs		SeNP (2,5, 5, 10, 20 mg/kg), healthy, arthritis, drug control (Lef)			Oral	14		Antioxidant enzymes, joint inflammation, biochemical index		[46]
RA	W		SeMetFa-NPs		SeMetFa nanoparticles (250, 500, and 750 µg/kg b.w.), healthy, RA, Prednisolone, CMC -vehicle control			-	21		Antioxidant enzymes, joint inflammation, inflammatory markers		[43]
RA	W		SeNPs		Control, RA, RA+SeNPs (250 and 500 µg/kg), RA+Celecoxib, RA + CA			IP	16		Antioxidant enzymes, joint inflammation		[44]
RA	W		CuO		Plant extract, low dose of NPs, high dose of NPs, healthy, arthritis			Oral	21		Antioxidant enzymes, joint inflammation, heamatological and biochemical index		[59]
RA	W		RR-CuONPs		RR-CuONPs (10 mg/kg), Diclofenac sodium (5 mg/kg), healthy, RA			IPL	28		Antioxidant enzymes, joint inflammation, spleen, thymus, hematological and biochemical indexing		[38]
RA	W		FA-AgNPs		HP (25 mg/kg) FA-AgNPs L-HP-NPs hybrid L-HP-FA-AgNPs (1 and 3 mg/kg), healthy, arthritis (CFA), sham (saline injection), drug control (MTX)			Oral	3 weeks		Antioxidant enzymes, joint inflammation, organ index		[54]
RA	W		AuSPION		AuC, AuSPION, Control (sham), RA (CIA + vehicle), MTX			IP	19		Antioxidant enzymes, joint inflammation, genotoxicity		[57]
RA	W		AuNGs		Control, CIA, MTX 0.25 mg/kg, AuNGs 20 µg/kg			IP	20		Antioxidant enzymes, inflammatory markers		[52]
RA	W		GX-AgNPs-BG, GX-AgNPs		BG, GX-AgNPs-BG, GX-AgNPs, INDO, and DEXA			Oral	14		Antioxidant enzymes, inflammatory markers, organ index, joint inflammation		[45]
RA	W		SeNPs		Healthy, RA, Prednisolone (10 mg/kg bv.w), Se nanoparticles (100 mg/kg b.w, 250 mg/kg b.w, 500 mg/kg b.w, 750 mg/kg b.w.)			Oral	21		Antioxidant enzymes, inflammatory markers, joint inflammation, targeting of the nanoparticles		[47]
RA	W		AgNPs		Sham, arthritis, MTX (0.4 mg/kg), AgNPs			Oral	14		Antioxidant enzymes		[42]
OA	SD		Ta-NH_2_ NPs		MIA + Ta-NH_2_ NPs group, MIA + CAT group, heatlhy, sham, arthritis (MIA)			IA	2–10 weeks		Intracellular ros, antioxidant enzymes, joint inflammation, biochemical index, biodistribution of drug		[53]
OA	SD		OHA/HA-ADH@SeNPs		Sham, PBS, OHA/HA-ADH, and OHA/HA-ADH@SeNPs			IA	7 weeks		Antioxidant enzymes, joint inflammation, biochemical index		[55]
OA	W		SaIONPs		SaIONPs, Sham (with saline injection), OA (MIA)			Oral	14		Antioxidant enzymes, inflammatory markers		[58]
OA	SD		nCe		Control, OA, nCe (250, 500, 1000, 2000 µg/mL)			local TMJ injection	1 h–14 days (in vitro), 10 days (in vivo)		Antioxidant enzymes, joint inflammation, ex-plant studies		[56]
OA	C57BL/6J		GaSe_x_		GaSe_x_ (20, 50 µg/mL), sham and arthritis model			IA	8 weeks		Antioxidant enzyme, joint inflammation, senescence		[51]
OA	-		CeO_2_		-			-	-		Inflammatory markers		[48]
OA	LEW		PEG-MnO_2_		Sham, PEG-MnO_2_ (5 mg/mL)			IA	6 weeks		Antioxidant enzymes, inflammatory markers, joint inflammation		[50]
OA	C57BL/6		Au@CeO_2_, Au@CeO_2_-CK2.1		Control, ACLT, ACLT + Laser, Au@CeO_2_, Au@CeO_2_-CK_2_.1, Au@CeO_2_ + Laser, Au@CeO_2_-CK2.1 + Laser			IA	8 weeks		Antioxidant enzymes, inflammatory markers, joint inflammation		[49]
OA	C57BL/6		miR/IrO_2_@ZIF-8, IrO_2_@ZIF-8		Sham, arthritis (DMM), DMM + ZIF-8, DMM+IrO_2_@ZIF-8, DMM + miR@ZIF-8, DMM + miR/IrO_2_@ZIF-8.			IA	4 weeks		Antioxidant enzymes, inflammatory markers, joint inflammation, biodistribution of the nanoparticles		[41]
OA	SD		Fe-Cur		OA, control, OA/Cur, OA/Fe-Cur			IA	4 weeks		Antioxidant enzymes, inflammatory markers, joint inflammation		[36]
OA	SD		CeO_x_, PtCuO_x_/CeO_2-x_,		Sham, OA, CeO_x_, PtCuO_x_/CeO_2-x_, PtCu0_x_/CeO_2-x_ + NIR			IA	4 and 8 weeks		Antioxidant enzymes, inflammatory markers, joint inflammation, biodistribution of the nanoparticles		[39]
OA	W		PEG-CeONPs, WY-PEG-CeO		Sham, ACLT, ACLT + PEG-CeONPs, ACLT + WY-PEG-CeO			IA	8 weeks		Antioxidant enzymes, inflammatory markers, joint inflammation, biodistribution of the nanoparticles		[35]
OA	-		CNP		-			-	-		Antioxidant enzymes, inflammatory markers, explant studies		[40]
OA	SD		BSA-SSUL-Ce		PBS, OA, BSA-SSUL-Ce, BSA-SUL, BSA, SUL, CNLs.			IA	4 weeks		Antioxidant enzymes, inflammatory markers, joint inflammation, biodistribution of nanoparticles		[37]

**Clinical study**													
**Registry**		**Intervention**		**NP-system**		**Disease**	**Joint**			**Outcome**		**Reason of exclusion**	**Ref.**
Cochrane Library		*Phyllanthus amarus* formulated NPs gel		Biogenic (plant-based) NPs		**OA**	**Knee**			Reduced pain and oxidative stress		Didn’t meet the inclusion criteria of the current study	[61]

**Identified patents related to NPs based antioxidant therapies**													
**Database**		**Patent No.**		**Intervention (NPs type)**		**Disease**	**Application**			**Relevance to included studies**			**Ref**
PATENTSCOPE		CN114146191		organic-inorganic hybrid AuNPs		OA	Collagen-II targeted antioxidant therapy			Not linked to included studies			[62]
PATENTSCOPE		CN119499278		Selenium-based hydrogel microspheres		OA	Multistage antioxidant treatment			Not linked to included studies			[63]

Albuminized ceria nanozymes loaded with SUL (BSA-SSUL-Ce), anterior cruciate ligament transection (ACLT), bergenin (BG), cartilage-targeting peptide WYRGRLGK (WY), celecoxib (CXB), cerium dioxide (CeO2), Cerium oxide nanoparticles (CNP, CeO2, CeONP), collagen-induced arthritis (CIA), complete Freund's adjuvant (CFA), copper oxide (CuO), coumaric acid (CA), destabilization of the medial meniscus (DMM), dexamethasone (DEXA), diclofenac (Diclo), female (F), gallium (Ga), gold (Au), gold-coated superparamagnetic iron oxide nanoparticle (AuSPION), gum xanthan (GX), hesperidin loaded hybrid lecithin-folic acid silver nanoparticles (L-HP-FA-AgNPs), hyaluronic acid-adipic acid dihydrazide (HA-ADH), hydroxychloroquine (HCQ), indomethacine (INDO), intra-articular (IA), intraperitoneal (IP), intra-planter (IPL), iron-curcumin (Fe-Cur), iridium oxide (IrO2), iron oxide (IO, FeO), leflunomide (LEF), Lewis rats (LEW), male (M), manganese dioxide (MnO2), metal (Mt), metal oxide (MO), methionine-folic acid (MetFa), methotrexate (MTX), microRNA (MiR), monosodium iodoacetate (MIA), nanoceria (nCe), near-infrared (NIR), oxidized hyaluronic acid (OHA), platinum (Pt), polyethylene glycol (PEG), prednisolone (PRED), Rabdosia rubescens (RR), salicylic acid-functionalized iron oxide nanoparticles (SaIONPs), selenium (Se), Sprague-Dawley rats (SD), tantalum (Ta), temporomandibular joint (TMJ), Wistar rats (W), zeolitic imidazolate framework-8 (ZIF-8)

Quantitative pooling of extracted data for a meta-analysis was hindered by methodological heterogeneity across the included studies. There was variation in NPs’ design (composition, formulation, and functionalization), dosing strategies, administration routes, and treatment duration. Apart from this, narrative synthesis and comprehensive tabulation of study characteristics enabled identification of consistent trends, mechanistic insights, and existing research gaps.

Our review identified that in RA studies, the most frequently used Mt-NPs were gold (Au), silver (Ag), and selenium (Se), whereas iron (Fe) and copper (Cu) were commonly applied in their MO forms. Although Se is a non-metal, its metalloid properties [64] support its inclusion alongside metals in this review.

**Fig. 2:**
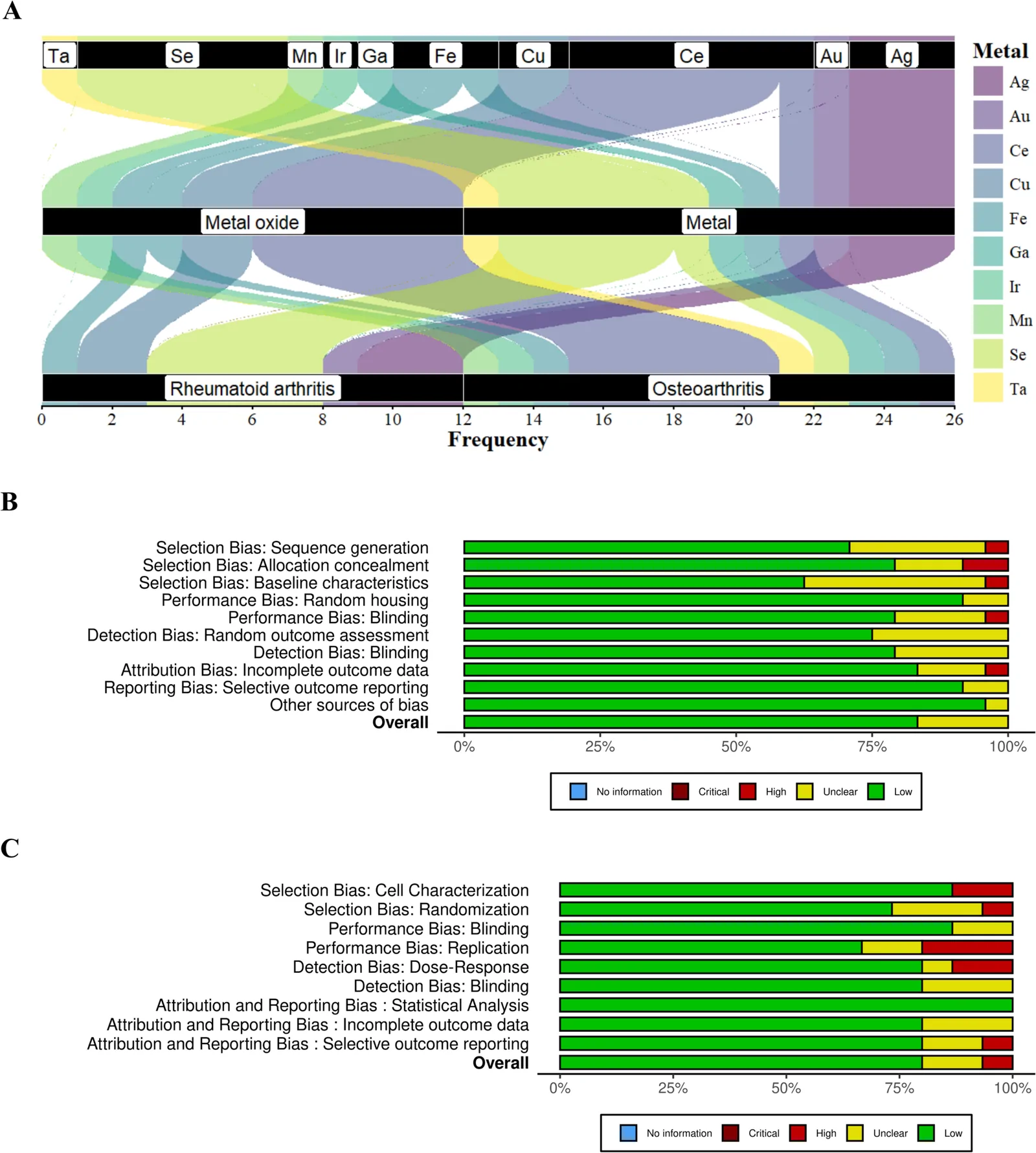
**(A)** Commonly used antioxidant NPs to treat RA and OA, risk of bias (ROB) analysis; **(B)** SYRCLE’s RoB tool for animal studies; **(C)** custom quality assessment check for in vitro studies

In contrast, a wider variety of Mt-NPs were employed in OA studies, including gallium (Ga), cerium (Ce), iron (Fe), tantalum (Ta), and selenium (Se), while the predominant MO-NPs were based on manganese (Mn), cerium (Ce), iron (Fe), and iridium (Ir). Overall, we observed that OA studies predominantly utilized MO-NPs, particularly those based on Ce, Fe, and Mn. In RA studies, the emphasis was more on Mt-NPs, especially Se, Ag, and Au, although Fe and Cu were still frequently observed in their oxide forms (Figure 2A).

### Risk of bias analysis

Of the 26 studies included in the review, 24 used animal models [35–39, 41–47, 49–60]. Of those, 20 were of high quality with low risk of bias, while the remaining four [43, 44, 54, 55] were rated as having a moderate or unclear quality (Figure 2B) due to incomplete information about the quantity, baseline characteristics, and study group of the animals used; undefined methodology; and selective reporting of the research results.

Altogether, 15 studies used in vitro models [35–37, 39–41, 45, 48–51, 53–56]; of these, 12 were assessed as high quality and two [45, 54] as moderate. One study [48] was determined to be of low quality with high risk of bias (Figure 2C) because of insufficient information about cell characteristics, study group randomization, replication, dose-response, and selective outcome reporting. Figure S1-2 shows the details of the assessment questions.

### Nanoparticle properties and administration

Reviewed studies included great heterogeneity in some key parameters such as size, zeta potential, surface engineering, dosage, and route of administration strategy, which influences biodistribution and cellular uptake of NPs. To understand the relationship between NPs design and therapeutic efficacy, the physicochemical properties of the evaluated nanoparticles were systematically compared in Table 2. The nanoparticles included elemental metals, metal oxides, and hybrid nanocomposites. In general NP sizes were mostly in range of 15–100 nm, which is generally acceptable for cellular uptake and tissue penetration. However, overall size distribution varied from ultrasmall NPs (IrO₂: 2.7 nm, Fe-Cur: 2.5 nm, and CeO₂: 5–6.8 nm) to larger systems (HA hydrogel-loaded CeO₂ 460 ± 20 nm, Tantalum NPs: 168.77 nm, hybrid Silver NP: 150–190 nm). Various surface-engineering strategies were used to improve stability, targeting ability, bioavailability, or therapeutic efficacy. Some of the NPs were hybrid nanocomposites (Au@CeO₂, Gold-coated SPIONs, PtCuO_x_-dispersed CeO₂). Other coating systems included various biopolymers for stabilization (PEGylation (polyethylene glycol), Polyvinylpyrrolidone (PVP), Gum xanthan, Dextran), targeting ligands (Folic acid coatings, Cartilage-targeting peptide (WYRGRLGK), Methionine-folic acid coating) and encapsulation (Hyaluronic acid and ZIF-8). These combinations resulted broad range of surface charges from −43.67 mV (BSA-SSUL-Ce) to +50.5 mV (Salicylic acid-functionalized iron oxide). Most common administration routes were intra-articular, oral, and intraperitoneal, where last two result to systemic exposure. In addition to intra-articular, also intraplantar and temporomandibular joint injections were used for local delivery. 

**Table 2:** Systematic comparison of physicochemical properties of the evaluated nanoparticles

Material	Form	Surface Modifications	Size (nm)/Zeta Potential (mV)	Biodistribution Studies			Therapeutic Studies			Ref
				Dosage	Route	Duration (Days)	Dosage	Route	Duration (Days)	
Se	Mt	-	47	2.5, 5, 10 and 20 mg/kg	Oral	14	2.5, 5, 10, 20 mg/kg	Oral	14	[60]
Se	Mt	-	43	2.5, 5, 10 and 20 mg/kg	Oral	14	2.5, 5, 10, 20 mg/kg	Oral	14	[46]
Se	Mt	Methionine-folic acid coating	50	250, 500 and 750 µg/kg	-	21	250, 500, 750 µg/kg	-	21	[43]
Se	Mt	-	68	0.25, 0.5 and 5 mg/kg	IP	16	250 and 500 µg/kg	IP	16	[44]
CuO	MO	-	186.8/−9.23	8 and 12 mg/kg	Oral	21	8 and 12 mg/kg	Oral	21	[59]
CuO	MO	*Rabdosia rebescens* formulated synthesis (RR-CuONPs)	30–90	10 mg/kg	IPL	28	10 mg/kg	IPL	28	[38]
Ag	Mt	FA surface modification, Hesperidin-loaded hybrid lecithin-folic acid silver nanoparticles	151.5 ± 1.71/−29.5 ± 0.21	1 and 3 mg/kg	Oral	21	1 and 3 mg/kg	Oral	21	[54]
Fe_3_O_4_	MO	Gold-coated superparamagnetic iron oxide nanoparticles (AuSPION)	18–22/−26.4	150 µg/kg	IP	19	150 µg/kg	IP	19	[57]
Au	Mt	-	15	20 µg/kg	IP	20	20 µg/kg daily no in vitro dose information	IP	20	[52]
Ag	Mt	Gum xanthan (GX) stabilized silver nanoparticles (GX-AgNPs) and GX stabilized AgNPs coated with Bergenin (GX-AgNPs-BG)	182.71 ± 1.04/−9.11 ± 1.80 (GX-AgNPs) 189.20 ± 2.09/−16.20 ± 0.40 (GX-AgNPs-BG)	0.5 and 1 mg/kg	Oral	7	BG (25 mg/kg), GX-AgNPs-BG (1 mg/kg), GX-AgNPs (1 mg/kg),	Oral	14	[45]
Se	Mt	Dextrin stabilized Se nanoparticles (SeNP)	64	5 mg/kg	Oral	28	10–100 µg/ml, 100, 250, 500, 750 µg/kg	Oral	21	[47]
Ag	Mt	Guggul (G)	50–70/−18.9 ± 1.8	100, 200 and 300 µg/kg	Oral	7	2 000 mg/kg p. o., 1.75 mg/kg, 100,200,300 µg/kg	Oral	14	[42]
Ta	Mt	NH_2_ modified	168.77 ± 8.04/32.27 ± 0.51	33–40 µg/kg	IA	28	0, 5, 10, 20, 50, 100,200, 500 µg/ml	IA	2–10 weeks	[53]
Se	Mt	Encapsulation in Oxidized Hyaluronic Acid (OHA) crosslinked with hyaluronic acid-adipic acid dihydrazide (HA-ADH)	-	10 µg/injection	IA	49	1, 2, 5, 10 and 100 µg/ml	IA	7 weeks	[55]
IO	MO	Salicylic acid-functionalized iron oxide nanoparticles (SaIONPs)	73/+50.5	0.67 mg/rat	Oral	14	1 ml daily	Oral	14	[58]
Ce	M	-	19.5	7.5, 15, 30 and 60 µg/injection	IA	10	in vitro (40, 80, 160 and 320 µg/ml), in vivo (250, 500, 1000, 2000 µg/mL (Single dose)	Local TMJ injection	1 h-14 days (in vitro), 10 days (in vivo)	[56]
GaSe_x_	Mt	GaSe_x_ nanoparticles with DSPEPEG5000 surface modification	17	20 and 50 µg/ml	IA	56	0, 12, 20, 25, 50, and 100 µg/ml	IA	8 weeks	[51]
CeO_2_	MO	Hyaluronic acid (HA) hydrogel loaded with PL and CeO_2_-NPs	460 ± 20/11 ± 2	-	-	-	DPPH: 10, 20, 40, 100, 200 g/ml. Hydrogel form: 5%w/v.	-	-	[48]
MnO_2_	MO	PEG-SVA conjugated	15/+52.12	100 µg/injection	IA	11	0, 5, 10, 20, 50, 100 and at 250 µg/mL (5 µg/ml after screening), 20 µl of 5 mg/ml	IA	6 weeks	[50]
Au@CeO_2_	MO	Au@CeO_2_-CK2.1	106	1.25 µg/injection	IA	56	25 µg/ml (50 µl-twice a week)	IA	8 weeks	[49]
IrO_2_	MO	miR/IrO_2_@ZIF-8 (PVP)	2.7/_2_0.4 ± 1.2 (IrO2), and 178 7 − 5.6 ± 0.8 (IrO2@ZIF-8)	5 mg/ml	IA	28	10, 50, 200 µg/ml. 5 mg/ml	IA	4 weeks	[41]
Fe-Cur	Mt	Poly(vinylpyrrolidone) (PVP)	2.5	20 mg/ml	IA	28	0, 6, 12, 25, 50, 100, 200 µg/ml (in vitro), 20 mg/kg	IA	4 weeks	[36]
CeO_2_	MO	Dispersion of Cu and Pt nanospheres (PtCuO_x_)	170	5 µg/injection	IA	28 OR 56	0, 20, 50, 100 µg/ml	IA	4 and 8 weeks	[39]
CeO_2_ (CeO)	MO	Polyethylene glycol-packed CeONPs (PEG-CeONPs), and cartilage-targeting peptide WYRGRLGK (WY-PEG-CeO)	CeONPs: 6.5, WY-PEG-CeO: 6.8	25 µg/injection	IA	56	0, 12.5, 25, 50, 100, and 200 µg/ml, 500 µg/ml (In vivo Ce concentration)	IA	8 weeks	[35]
CeO_2_ (CNP)	MO	-	5	No in vivo assay performed	-	-	40, 80, 160, 320 µg/ml	-	-	[40]
Ce	Mt	BSA-SSUL-Ce	17.79/−43.67	50 µl/injection	IA	35	2.5, 10, 20, 40, 50, 100, 200 µM, 50 µl	IA	4 weeks	[37]

Albuminized ceria nanozymes loaded with SUL (BSA-SSUL-Ce), acrylate-polyethylene glycol succinimidyl valerate (PEG–SVA), cerium oxide nanoparticles (CNP, CeO, CeO2), copper oxide (CuO), gallium (Ga), selenium (Se), iron oxide (IO, Fe3O4), intra-articular (IA), intraperitoneal (IP), intra-planter (IPL)

### Safety, biodistribution, and toxicity aspects

**Fig. 3:**
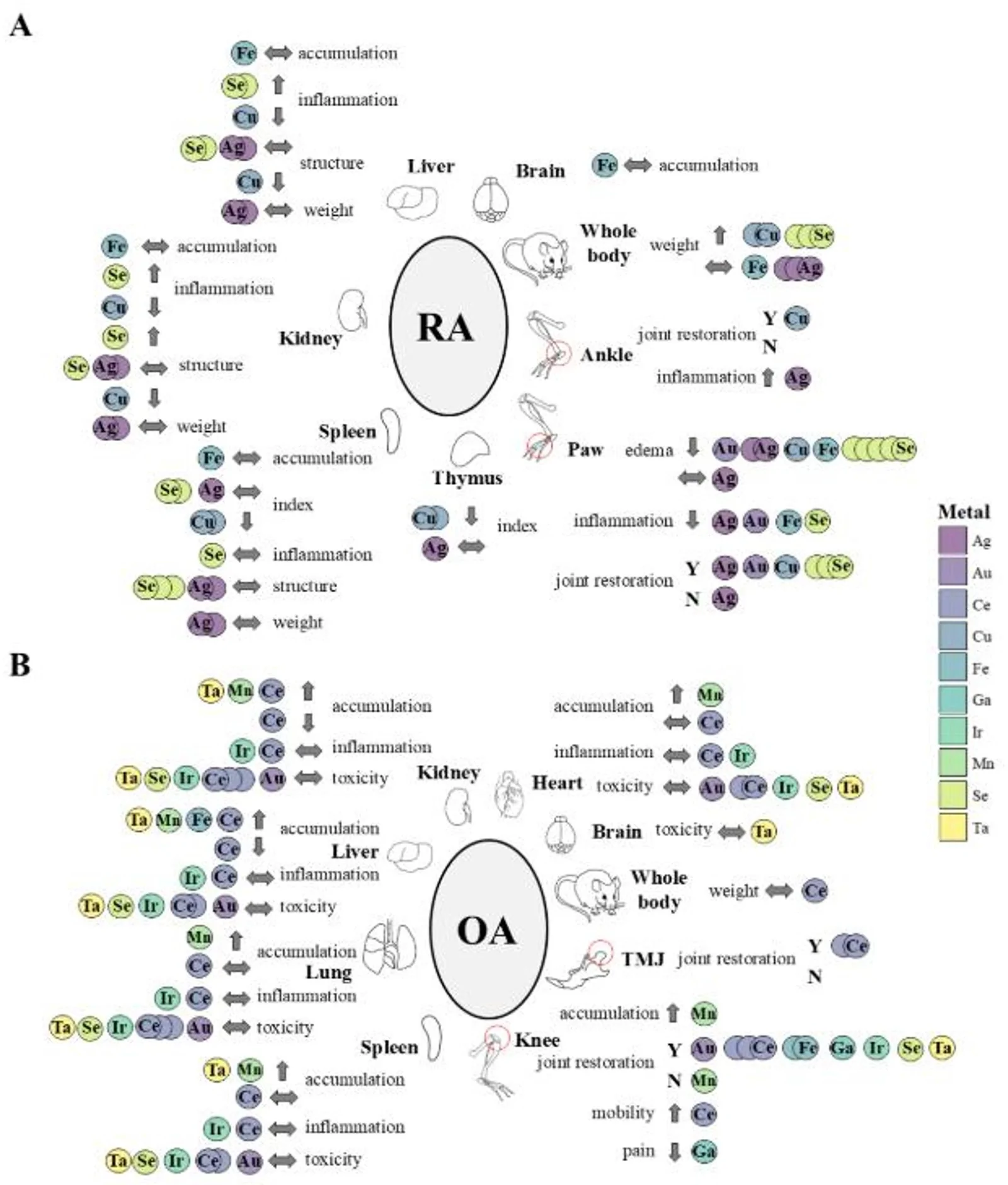
Graphical presentation of the biodistribution and joint specific anti-arthritic outcomes of NPs in (**A**) RA and (**B**) OA

Figure 3, Table S2, and Table S3 illustrate the biodistribution, toxicity, and anti-arthritic outcomes following the NP treatment from the reviewed studies. In vivo studies of RA revealed no major organ toxicity (e.g., liver, kidney, or spleen) following oral administration of SeNPs at doses of 250 µg/kg–5 mg/kg [43, 44, 46, 47, 60]. AgNPs at dose of 1.75 mg/kg was found to be lethal [42] unless additional stabilization (hesperidin hybrid lecithin-folic acid, bergenin loaded gum xanthan) was included [45, 54]. Interestingly, CuONPs potentially decreased organ index at doses of 8–12 mg/kg, suggesting that these NPs may prevent tissue damage [59]. Four studies evaluated liver and renal function [38, 46, 59, 60] and mostly saw no changes compared to healthy controls, except in higher concentrations of SeNPs (increase in urea [46], increase in Alk [60]) and CuONPs (increase in ALT and AST) [59]. Two studies additionally assessed haematological parameters, including red blood cells (RBCs), white blood cells (WBCs), platelets, and haemoglobin (Hb) [38, 59], but only reported changes relative to disease control and lacked an untreated control group. Additionally, one study [43] evaluated the haemolytic effect of SeMetFaNPs and found 15% haemolysis at the highest dose (200 µg/mL). In vitro cytotoxicity was assessed only for SeNPs in fibroblasts [47], and coating was found to increase short-term viability up to 75% versus 5% (uncoated) at the same dose.

In OA, generally, no structural changes or accumulation of inflammatory cells in major organs (mostly heart, kidney, liver, lung, and spleen) were detected in any NP type or dose, even though noticeable tissue accumulation of NPs was present. Studies [37, 53, 55] assessed biochemical indices of liver and kidney function and reported no significant differences compared with healthy controls. In three studies [37, 39, 41] only minimal effects (< 5% haemolysis) were observed even at the highest tested concentrations. The most comprehensive blood cell panel on Ta-NH_2_NPs [53] did not show any changes, indicating good biocompatibility. Most studies evaluating OA in vitro assessed toxicity in chondrocytes or chondrosarcoma cells. In general, reduced viability was detected only at the highest doses of some SeNPs [55] and CeNPs [35, 40, 49, 56].

In OA, the most common administration route was intra-articular injection, whereas oral administration or intraperitoneal injections were used in RA studies. In addition, dosages and dosing intervals varied significantly between all studies. Blood parameters were evaluated in only seven of the 29 included studies. In vitro testing with cell lines was performed in most OA studies, but in very few RA studies. Moreover, the in vitro tests were short (a maximum of 48 h), and the in vivo toxicity tests lasted a maximum of 28 days (RA) or 56 days (OA) for each tested NP. Thus, long-term efficacy and safety remain to be estimated.

While the short-term safety profile of antioxidant NPs in RA and OA models appears favorable (with maximum follow-ups of only 28 and 56 days, respectively), critical knowledge gaps remain regarding their long-term toxicity and accumulation in the body and specific tissues. For example, Mt- (e.g., Au, Ag) and heavy MO- (e.g., CeO_2_, MnO_2_) NPs are often non-biodegradable. Consequently, even following localized intra-articular injection for OA, these NPs can gradually enter systemic circulation and accumulate in the reticuloendothelial system, primarily the liver, spleen, and kidneys. Over extended periods, this continuous accumulation may induce chronic oxidative stress, paradoxically countering their initial antioxidant benefits leading to hepatotoxicity, nephrotoxicity, and structural organ damage. Furthermore, the long-term immunological responses to these nano-formulations remain under-investigated. The unique physicochemical properties of NPs, including their surface coatings (e.g., PEG, folic acid), can act as haptens or adjuvants, potentially triggering delayed hypersensitivity reactions, complement activation, or chronic macrophage polarization toward pro-inflammatory phenotypes. In autoimmune conditions like RA, where the immune system is already dysregulated, the unintentional immunogenicity of accumulated NPs could exacerbate the underlying pathology over time. Therefore, future studies must prioritize long-term pharmacokinetic profiling (ideally over 6 months) and comprehensive immunological evaluations to ensure that the persistent tissue presence of these NPs does not elicit latent autoimmune or toxicological cascades.

### Role of Antioxidant NPs on RA

Among the 12 studies (Table 1), the most common method of RA induction was complete Freund’s adjuvant (CFA) in Wistar rats [38, 43–45, 47, 54, 59]. Collagen type II was used to induce arthritis (CIA) in Balb/c mice [46, 60] and Wistar rats [52, 57], while adjuvant-induced arthritis (AIA) was used in Wistar rats [42].

SeNPs and AgNPs were frequently used in the included studies to treat RA. Different types of surface functionalization of NPs were employed in studies of RA to improve their bioactivity, stability, and tissue specificity. Most of the studies used unmodified SeNPs [44, 46, 47, 60], whereas one study employed methionine-folic acid-modified formulations to improve their bioactivity, stability, and tissue specificity [43].

Similarly, one study [42] used unmodified AgNPs while Jabri et al. [54] applied folic acid and Rao et al. [45] applied Gum xanthan (GX) and Bergenin modifications. Babar et al. [59] studied unmodified CuONPs, whereas Zhang et al. [38] used *Rabdosia rebescens*-formulated CuONPs (RR-CuONPs). Khan et al. [52], and Carneiro et al. [57] employed Au-modified IONPs. These NPs were investigated in animal models at various concentrations (250 µg/kg–500 mg/kg) for treatment durations of 14–28 days.

**Fig. 4:**
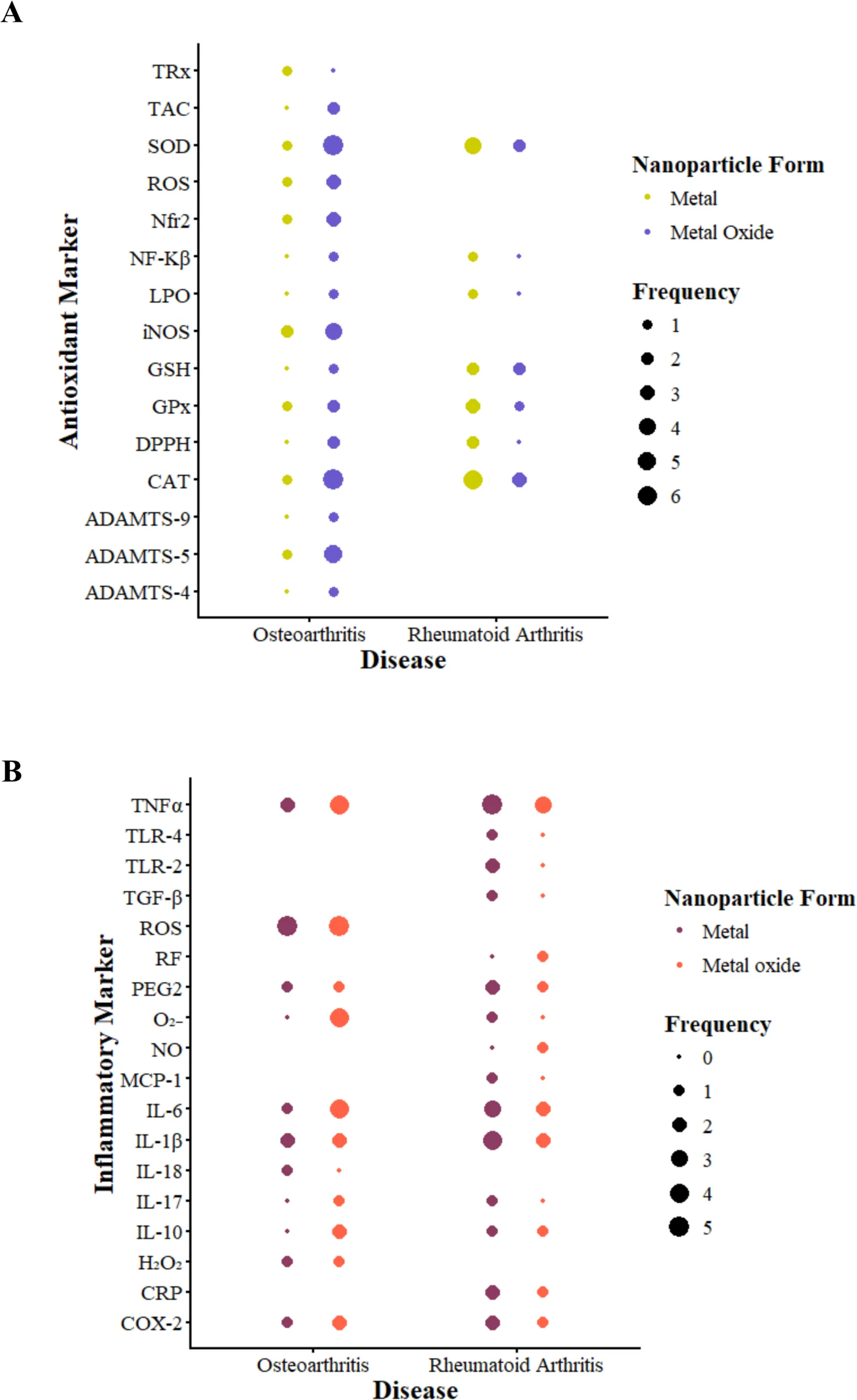
Markers used to evaluate the antioxidant & anti-inflammatory activities of NPs in the included studies: **(A)** Antioxidant markers; **(B)** Inflammatory markers. Number of studies with the specific anti-inflammatory/anti-oxidant markers (frequency/circles), intracellular radical scavenging activities (ROS)

The most frequently examined antioxidant enzymes were catalase (CAT), superoxide dismutase (SOD), glutathione peroxidase (GPx), and glutathione (GSH). Ag, Se, and CuO NP treatments reportedly increased the CAT [38, 42–44, 46, 47, 59, 60] and SOD enzymatic activities [38, 42–44, 47, 59] while the SeNPs treatment increased the GPx activity [43, 44, 47]. Se, Au, CuO, and AuSPION NPs reportedly increased the GSH activity [38, 44, 52, 57] while no significant changes in the levels of CAT, SOD, and GPx followed AuSPION NPs [57] (Figure 4A).

The key pro-inflammatory cytokines evaluated across these studies were TNF-α, IL-1β, and IL-6 compared to arthritic controls. CuO, Se, Ag, Au, and AuSPION NPs treatments reportedly decreased the levels of TNF-α, IL-1β, and IL-6 [38, 43–45, 52, 54, 57, 59] (Figure 4B).

Table 3 presents the information on the markers used to evaluate the antioxidant, anti-inflammatory, and anti-arthritic activities of NPs in the included RA studies. Across the studies, SeNPs and CuONPs with various surface modifications, different routes of administration, and treatment durations resulted in increased activities of antioxidant enzymes and reduced levels of pro-inflammatory enzymes. Most studies reported increases in ROS scavenging, antioxidant enzyme activities (SOD, CAT, GPx, GSH), and redox-regulating gene expression (RANK, RANKL, ECSOD, GPx2, PEDX1, MnSOD, TLR-2, Cu/ZnSOD, RORγt, MMP2/9). Treatment led to increased anti-inflammatory cytokines, decreased pro-inflammatory cytokines, and modulation of macrophage polarization, along with joint restoration, indicating strong anti-osteoclastic effects, and likely reflecting formulation-specific properties.

**Table 3:** Markers used to evaluate the antioxidant, anti-inflammatory, and anti-arthritic activities of NPs in the included studies

Model	Nanoparticles	Inflammatory Markers	Antioxidant enzymes	Joints	Outcomes		Ref.
					Antioxidant activities	Joint specific outcome	
RA	SeNPs	-	DPPH, CAT	Hind paw	↑ Antioxidant enzymes	↓ Edema & dose-dependent effects on joint restoration	[60]
RA	SeNPs	-	DPPH, CAT	Paw	↑ Antioxidant enzymes	↓ Edema & joint restoration	[46]
RA	SeMetFa-NPs	TNF-α, CRP, PGE2	SOD, CAT, GPx	Hind paw	Immuno-redox modulation	↓ Edema	[43]
RA	SeNPs	COX2, TNF-a, IL-1b, IL-6, MCP-1, TBARS	GSH, GPx, SOD, CAT, MnSOD, Cu/ZnSOD, ECSOD, GPX-1, GPX-2, CAT	Paw	Immuno-redox modulation	↓ Edema & dose-dependent effects on joint restoration	[44]
RA	CuO	TNF-α, IL-6, CRP, RF	SOD, CAT	Ankle	Immuno-redox modulation	↓ Edema & joint restoration	[59]
RA	RR-CuONPs	COX2, IL-10, IL-6, TNF-a, IL-1b, PGE2, TBARS	SOD, CAT, GSH	Paw	Immuno-redox modulation	↓ Edema & joint restoration	[38]
RA	FA-AgNPs	pro-inflammatory cytokines in macrophages & cultured splenocytes. Synovium: RANKL, OPG, MMP2, MMP9 Spleen: RANK, RANKL, RORγt	-	Right hind paw & secondary ankle joint	Immuno modulation	Paw swelling (↔) & no joint restoration	[54]
RA	AuSPION	TNF-α and IL-1β, plasmatic F2-isoprostanes (F2-IsoPs), plasmatic nitric oxide (NO) levels	GSH, GSH-Px, CAT, genotoxicity	Paw (right ankle)	Immuno modulation & moderate antioxidant activities	↓ Edema & inflammation	[57]
RA	AuNGs	COX2, TNF-α, IL-1β	LPO, GSH, NF-κB	Paw joint	Immuno-redox modulation	↓ Edema & joint restoration	[52]
RA	GX-AgNPs-BG, GX-AgNPs	IL-1β, IL-6, TNF-α, NO, PO, TLR-2, TLR-4, O2- production		Knee joint, right ankle joints of the hind limbs	GX-AgNPs-BG: immuno modulation GX-AgNPs shows no effect	↓ Edema & joint restoration	[45]
RA	SeNPs	CRP, PGE2	antioxidant effect on DNA, SOD, CAT, GPx,	Paw	Immuno-redox modulation	↓ Edema & inflammation	[47]
RA	AgNPs	-	SOD, CAT	Paw	↑ Antioxidant enzymes	↓ Edema & inflammation	[42]
OA	Ta-NH_2_ NPs	intracellular ROS, H_2_O_2_; ACAN, SOX9, COL-I, COL-II, & MMP13	RUNX-2, iNOS, ADAMTS5	Knee joint	Immuno-redox modulation	Joint restoration	[53]
OA	OHA/HA-ADH@SeNPs	IL-1β, TNF-α, intracellular ROS; MMP13, COL-II	GPx, caspase-3, thioredoxin reductase	Knee cartilage	Immuno-redox modulation	Joint restoration	[55]
OA	SaIONPs	TNF-α & NO	total antioxidant capacity (TAC), malondialdehyde (MDA), CAT, and reduced glutathione (GSH)	Knee	Immuno modulation & no redox activity	Mild effects on joint restoration	[58]
OA	nCe	Pro-inflammatory genes (TNF-α and IL-1β), anti-inflammatory genes (IL-10 CD163), COX2, intracellular ROS	SOD, DPPH, PGE2, the expression of iNOS	TMJ condyles	Immuno-redox modulation	Joint restoration	[56]
OA	GaSex	intracellular ROS, and ECM secretion of extracellular matrix by chondrocytes, senescence-associated -galactosidase (SA--Gal), intracellular GSH, Iron, Ferrous Ion, as well as MDA content measurement	-	Knee joint, human osteochondral plugs from knee	Immuno modulation	↓ Pain & joint restoration	[51]
OA	CeO_2_	COL-II, ACAN, COL-X, MMP13	radical scavenging activity, ADAMTS5, ADAMTS9	-	Redox modulation	-	[48]
OA	PEG-MnO_2_	NO, H_2_O_2_, TNF-α, IL-10, MMP1, MMP13, GAGs	iNOS, ADAMTS5, ADAMTS4, GPX, MnSOD, ECSOD, CAT, NRF2, KEAP1, HMOX1, NQO1, TXN1, PRDX1, SREBP1, PPARα, CPT2, ACACA1,	Cartilage explant & knee joint	Immuno-redox modulation	NPs accumulation & no joint restoration	[50]
OA	Au@CeO_2_, Au@CeO_2_-CK2.1	intracellular ROS, COL-II, SOX9, ACAN, MMP3, MMP13,MFN1, OPA1, DRP1, FIS1, mitochondrial ROS, mitochondrial membrane potential	SOD, GPx, CAT, SOD1, CAT, GPx1, Nrf2, ADAMTS5, TAC	Knee joint	Immuno-redox modulation	Joint restoration	[49]
OA	miR/IrO_2_@ZIF-8, IrO_2_@ZIF-8	IL-6, intracellular ROS, MMP13, COL-II, ACAN, mitochondrial membrane potential	CAT, SOD, ADAMSTS-5, ABTS, lipid peroxidation	Knee joint	Immuno-redox modulation	Joint restoration	[41]
OA	Fe-Cur	COX2, NLRP3, caspase-1, IL-18, intracellular ROS, MMPs, COL-II, ACAN	Nrf2(HO-1, NQO1), Scavenging activities (RNS, ROS, OH), iNOS	Knee joint	Immuno-redox modulation	Joint restoration	[36]
OA	CeO_x_, PtCuO_x_/CeO_2-x_,	IL-6, TNF-α, IL-1β, IL-17, intracellular ROS, NO, mitochondrial membrane potential, MMP13, COL-II, ACAN	SOD/CAT like enzymatic activity (O_2-_, H_2_O_2_, ·OH, and DPPH), ROS/Rac-1/NF-κkB (Rac-1, p-p65/p65), iNOS	Knee joint	Immuno-redox modulation	↑ Mobility & joint restoration	[39]
OA	PEG-CeONPs, WY-PEG-CeO	IL6, p-JNK, JNK, p-P38, P38, intracellular ROS, P16, P21, MMP3, MMP13, p-PI3K, PI3K, p-AKT, AKT, Ki-67, ACAN, COL-II	SOD/CAT, ADAMTS5, radical scavenging activities	Knee joint, porcine femur	Immuno-redox modulation	Joint restoration	[35]
OA	CNP	COX2, IL-6, intracellular ROS, NO, O_2-_, ACAN, COL-I, COL-II, MMP13	SOD/CAT, ADAMST4, iNOS, Nrf2-siRNA, Nrf2, HO-1,	Temporomandibular joint	Immuno-redox modulation	Joint restoration	[40]
OA	BSA-SSUL-Ce	Intracelullar ROS, HIF-1α, IL-1β, IL-6, TNF-α, O_2-_, SOX9, ACAN, MMP3, MMP9	SOD, CAT	Knee joint	Immuno-redox modulation	Joint restoration	[37]

2,2-diphenyl-1-picrylhydrazyl (DPPH), 2,2′-azino-bis(3-ethylbenzothiazoline-6-sulfonicacid) (ABTS), a disintegrin and metalloproteinase with thrombospondin motifs (ADAMTS), acetyl-CoA carboxylase alpha 1 (ACACA1), aggrecan (ACAN),albuminized ceria nanozymes loaded with SUL (BSA-SSUL-Ce), bergenin (GX), carnitine palmitoyltransferase 2 (CPT2), catalase (CAT), cartilage-targetingpeptide WYRGRLGK (WY), cerium oxide nanoparticles (CeO2, CeOx, CNP), c-Jun N-terminal kinase (JNK), cluster of differentiation 163 (CD163), copper oxide(CuO), copper/zinc superoxide dismutase (Cu/ZnSOD), C-reactive protein (CRP), cyclin-dependent kinase inhibitor p16 (P16), cyclin-dependent kinase inhibitorp21 (P21), cyclooxygenase-2 (COX2), dynamin-related protein 1 (DRP1), extracellular superoxide dismutase (ECSOD), F2-isoprostanes (F2-IsoPs), folic acid-modified silver nanoparticles (FA-AgNPs), gallium selenide nanoparticles (GaSex), glutathione (GSH), glutathione peroxidase (GPx, GSH-Px, GPX),glycosaminoglycans (GAGs), gold (Au), gold-coated superparamagnetic iron oxide nanoparticle (AuSPION), gum xanthan (GX), heme oxygenase-1 (HO-1,HMOX1), hydrogen peroxide (H2O2), hydroxyl radical (·OH), hyaluronic acid-adipic acid dihydrazide (HA-ADH), hypoxia-inducible factor 1-alpha (HIF-1α),inducible nitric oxide synthase (iNOS), interleukin (IL), iridium oxide (IrO2), Kelch-like ECH-associated protein 1 (KEAP1), Ki-67 proliferation antigen (Ki-67),lipid peroxidation (LPO), malondialdehyde (MDA), manganese superoxide dismutase (MnSOD), matrix metalloproteinase (MMP), methionine-folic acid (MetFa), microRNA (MiR), mitochondrial fission 1 protein (FIS1), mitofusin 1 (MFN1), monocyte chemoattractant protein-1 (MCP-1), NAD(P)H quinone dehydrogenase 1 (NQO1), nanoceria (nCe), NLR family pyrin domain-containing protein 3 (NLRP3), nitric oxide (NO), nuclear factor erythroid 2-related factor 2 (Nrf2), nuclear factor kappa B (NF-κB), optic atrophy 1 (OPA1), osteoprotegerin (OPG), oxidized hyaluronic acid (OHA), p38 mitogen-activated protein kinase (P38), peroxiredoxin 1 (PRDX1), peroxisome proliferator-activated receptor alpha (PPARα), phosphoinositide 3-kinase (PI3K), polyethylene glycol (PEG), prostaglandin E2 (PGE2), protein kinase B (AKT), Rabdosia rubescens (RR), Ras-related C3 botulinum toxin substrate 1 (Rac-1), reactive nitrogen species (RNS), reactive oxygen species (ROS), receptor activator of nuclear factor kappa-B (RANK), receptor activator of nuclear factor kappa-B ligand (RANKL), retinoic acid receptorrelated orphan receptor gamma t (RORγt), rheumatoid factor (RF), runt-related transcription factor 2 (RUNX-2), salicylic acid-functionalized iron oxide nanoparticles (SaIONPs), selenium (Se), senescence-associated beta-galactosidase (SA-β-Gal), silver (Ag), small interfering RNA (siRNA), SRY-Box transcription factor 9 (SOX9), sterol regulatory element-binding protein 1 (SREBP1), superoxide anion (O2-), superoxide dismutase (SOD), tantalum (Ta), thioredoxin 1 (TXN1), thiobarbituric acid reactive substances (TBARS), toll-like receptor (TLR), total antioxidant capacity (TAC), tumor necrosis factor alpha (TNF-α, TNF-a), zeolitic imidazolate framework-8 (ZIF-8), increase/upregulate (↑), decrease/downregulate (↓)

Only FA-AgNPs treatment reduced inflammatory markers, without altering joint edema. The findings contrast sharply with the biogenic GX-AgNPs [45], which, via the same administration route, produced significant anti-inflammatory effects and marked histopathological improvement within 14 days. The enhanced joint-protective effects, marked by reduced oxidative stress through interference with the NADPH oxidase metabolic pathway, likely reflect formulation-specific properties.

Conflicting antioxidant responses to AuNPs indicate strong dose-dependent effects. IP administration of colloidal AuNPs administered at a higher fixed-dose (150 µg/kg) at 2-day intervals for 19 days reduced GSH and increased NO to RA levels, whereas Au-coated AuSPION NPs increased GSH with decreasing NO [57]. In contrast, AuNPs given at a lower, weight-adjusted dose of 20 µg/kg daily for 20 days increased GSH, indicating that AuNPs may exert antioxidant effects depending on exposure intensity. AuNPs exert anti-inflammatory and chondroprotective effects by inhibiting NF-κB activation and modulating other inflammatory modulators, such as COX2, IL-1, iNOS, TNF-, IL-6, and ROS [52].

### Role of antioxidant NPs on OA

Among the 14 studies, the most common method to induce OA was the destabilization of the medial meniscus (DMM) in Sprague–Dawley rats [36, 55], C57BL/6 [41], and C57BL/6J mice [51]. Anterior cruciate ligament transection (ACLT) was performed in Sprague–Dawley [39] and Wistar rats [35] and C57BL/6 mice [49].

Collagenase-induced osteoarthritis (CIOA) [37] and CFA [56] were used in Sprague–Dawley rats. Furthermore, monoiodoacetate-induced arthritis (MIA) was observed in Sprague–Dawley rats [53] and Wistar rats [58]. Moreover, Kumar et al. [50] used Lewis rats for the biodistribution studies only; Xiong et al. [40] employed temporomandibular joint explants from Sprague–Dawley rats; and Li et al.’s [48] study reported only in vitro studies. Table 1 summarizes the included studies.

In OA, various nanoformulations (NPs, nanozymes, or nanocarriers) were used with different surface functionalizations to tune their targeting properties. CeO_2_ NPs were frequently used to treat OA across the included studies. Several studies used NPs in unmodified forms [40, 56], whereas others employed modified formulations to enhance their bioactivity, stability, and tissue specificity. The surface modifications used in these studies included polyethylene glycol (PEG) [35, 50], cartilage-targeting peptide WYRGRLGK (WY-PEG-CeO) [35], platelet lysate, hyaluronic acid-based hydrogel [48], salicylic acid-functionalization [58], NH_2_ [53] and Fe-CurNPs modified with poly(vinylpyrrolidone) [36] respectively.

Similarly, Yang et al. [39] embedded copper and platinum nanospheres in a CeO_2_ framework, while another study [49] prepared Au@CeO_2_, a yolk-shell nanostructure. Wu et al. [41] developed IrONPs, encapsulated them into zeolitic imidazolate framework-8, and loaded them with Cy5-antagomiR-181 (miR/IrO_2_@ZIF-8).

Hu et al. [55] employed SeNPs encapsulated in oxidized hyaluronic acid crosslinked with hyaluronic acid-adipic acid dihydrazide (OHA/HA-ADH@SeNPs), while Kong et al. [51] employed Ga and SeNPs with DSPEPEG5000 surface modification. Zhou et al. [37] developed albuminized cerium nanozymes loaded with sulindac (BSA-SSUL-Ce) to increase the anti-inflammatory properties. The above-mentioned formulations of NPs were tested in animal models of OA at various concentrations (1 µg/mL to 20 mg/mL) for varying treatment durations from ten days to ten weeks.

The most common antioxidant markers evaluated across the OA studies were SOD, CAT, inducible nitric oxide synthase (iNOS), a disintegrin and metalloproteinase with thrombospondin motifs 5 (ADAMTS5), nuclear factor erythroid 2-related factor 2 (Nrf2), and the radical scavenging activities. The administration of Ce, CeO, and IrO NPs reportedly increased SOD activity [35, 37, 39–41, 49], whereas a decrease was observed after treatment with Ce and MnO_2_ NPs [50, 56]. CeO, Ce, and IrO NPs treatments reportedly increased CAT enzymatic activity [35, 37, 39–41, 49], whereas MnO_2_ NPs decreased it [50]. No significant effect was observed with SaIONP treatment, which might be attributed to its oral route of administration, as such hinders the targeted effects [58]. Fe, CeO, Ce, and Ta NPs treatment reduced iNOS levels [36, 39, 40, 53, 56]. CeO, IrO, MnO_2_, and Ta NPs also reduced ADAMTS5 expression [39, 41, 48–50, 53]. Nrf2 expression increased following Fe [36] and CeO NPs treatments [40, 49], while MnO_2_ NPs had no significant effect on iNOS or Nrf2 expression [50]. Additionally, Fe, CeO, and Ta NPs demonstrated dose-dependent improvements in radical scavenging activity [35, 36, 48, 53] (Figure 4A).

In OA studies, the most frequently examined inflammatory markers were the intracellular ROS, TNF-α, IL-6, IL-1β, and NO. Studies evaluating the effects of CeO, Ce, IrO, Ga, Ta, and Se NPs reported reduced intracellular ROS levels [35–37, 40, 41, 49, 51, 53, 55, 56]. Similarly, MnO_2_, Ce, CeO, Se, and SaIONPs treatment decreased TNF-α [37, 39, 50, 55, 56, 58], while IrO, Ce, and CeO NPs reduced IL-6 [35, 37, 39–41]. For NO, reductions were observed following CeO, MnO2, and SaIONPs [39, 40, 50, 58].

Ce and Se NPs reduced IL-1β [37, 55, 56], although one study reported an increase in IL-1β following CeONPs administration compared to the negative control [39] (Figure 3B). Findings align with those of Xue et al. [65], who reported that the wound-healing properties of CeO_2_ NPs were evidenced by enhanced ROS scavenging activities, modulation of the Nrf2 and NF-κB pathways, and CAT and SOD-like activities. Although the therapeutic contexts differ, both highlight the biological effects of CeO_2_ NPs and underscore the importance of NP formulations and dosages for optimizing efficacy and safety.

Most studies on OA used the animal knee joint to assess the effects of NPs. Nine studies reported preventing joint damage, mostly as indicated by reduced OARSI scores. One study by Bică et al. [58] reported mild joint restoration after SaIONPs treatment, while another [50] observed no significant effect on the knee joint histology with MnO_2_ NPs treatment. Furthermore, there are improved functional outcomes, including reduced pain [51] and improved mobility [39]. Moreover, two studies [40, 56] reported temporomandibular joint (TMJ) restoration.

Table 3 summarizes the antioxidant, anti-inflammatory, and anti-arthritic markers evaluated in these OA studies. Across the studies, IA administered unmodified CeNPs for ten days [56], BSA-SSUL-Ce formulated nanozyme for four weeks [37], CeO_2_NPs with varying surface modifications and treatment durations exert strong antioxidant effects in OA by increasing the activities of antioxidant enzymes, reducing apoptosis, catabolic factors, and inflammation, scavenging ROS, promoting cartilage protection and tissue regeneration, likely reflecting formulation-specific properties.

PEG-modified MnO_2_ NPs [50] efficiently penetrate cartilage explants and are internalized by chondrocytes without affecting matrix integrity and cell viability. They protect cartilage from cytokine-induced degeneration by decreasing apoptosis, NO production, and GAG loss, and suppressing the expression of catabolic enzymes (MMP1, MMP13, ADAMTS5), while maintaining aggrecan and collagen-II. These NPs also normalized antioxidant and redox-regulating gene expression (NRF2, KEAP1, ECSOD, GPx, PEDX1, MnSOD, TXN1, HMOX1) and mitigated the IL-1β-induced redox imbalance. PEG-coated MnO_2_ NPs demonstrate joint retention for at least seven days, selective cartilage localization, minimal off-target accumulation, chondrocyte uptake with no long-term joint toxicity, and supported their biocompatibility and chondroprotective efficacy. The efficient penetration, enhanced chondroprotective effects, and prolonged joint retention likely reflect size- and formulation-specific properties.

### Summary of key findings and mechanistic interpretation

The findings of the reviewed studies suggest that NP-based therapies hold promise as potential therapeutic strategies to manage RA and OA by modulating antioxidant and anti-inflammatory pathways while contributing to joint restoration. However, because the reviewed in vivo studies were relatively short-term (≤ 56 days) and involved limited toxicity evaluations, long-term safety and overall efficacy remain to be fully established.

Cartilage has a mesh of fibers (approx. ~50–60 nm) with anionic proteoglycans spaced (~20 nm) between side chains. Hence, thorough size optimization is critical to maximize efficacy while ensuring safety in joint-targeted therapies. Across the studies, the size of Mt- and MO-NPs significantly affects their therapeutic activities in RA and OA. Smaller NPs tend to have higher intracellular uptake, deeper tissue penetration, longer joint retention, and stronger antioxidant and anti-inflammatory effects. While larger NPs have poor intracellular penetration and uptake, they tend to accumulate on the cell surface and are rapidly cleared from the body.

Furthermore, Aurantii fructus immaturus carbonisata-derived carbon dots (AFIC-CDs) treatment significantly reduced monosodium urate (MSU)-induced inflammation, as evidenced by decreased joint swelling and suppressed inflammatory markers, highlighting their strong potential to manage hyperuricemia and gouty arthritis (GA), as Wang et al. [66]. Another study by Zhang et al. [67] engineered an IL-1 receptor antagonist chimera that forms PEG-induced protein NPs with significantly enhanced bioavailability and anti-inflammatory effects in gouty arthritis, indicating a broader therapeutic platform for GA and other inflammatory diseases.

Collectively, the findings suggest that NP-based therapeutics may enhance treatment outcomes by targeting disease-specific tissues, mitigating oxidative stress, and modulating inflammatory pathways. Given the unique pathophysiology of RA and OA, the mechanism of action appears to differ in each condition. In RA models, they primarily modulate immune responses and suppress inflammatory pathways; in OA models, they exhibit chondroprotective effects by mitigating oxidative stress and preserving cartilage integrity. Such disease-specific actions highlight the potential to tailor NP-based interventions for targeted and efficient treatment.

Detailed extracted information from all the reviewed studies, including the main summary, the markers used to evaluate the antioxidant and anti-inflammatory activities of NPs, and their biodistribution and anti-arthritic activities in joints and major organs, is provided in the supplementary file (Table S3-5). Although all included studies were preclinical, an extended search was conducted to assess the translational potential of NP-based antioxidant therapies. A patent database analysis (PATENTSCOPE) identified two patents related to Au- [62] and Se-based [63] NP systems for OA; however, neither corresponds to the included studies.

Notably, one clinical trial was identified [61] in the Cochrane Central Register of Controlled Trials. The study tested phonophoresis (ultrasound-assisted), the *Phyllanthus amarus*-formulated NPs gel in knee OA; the results showed reduced pain and inflammation. Although this study did not meet the inclusion criteria, it illustrates early efforts to translate NP-based therapies into clinical settings. Collectively, these findings highlight a clear translational gap between pre-clinical NP research and clinical applications. Initiating clinical trials with these specific antioxidant NPs presents several distinct challenges. First, the lack of long-term in vivo safety and pharmacokinetic data is an important regulatory hurdle. While short-term studies show minimal acute toxicity, the long-term accumulation of non-biodegradable inorganic NPs (such as Au, Ce, and Ag) in organs such as the liver and spleen remains a critical safety concern for human application. Second, there are significant manufacturing challenges; scaling up the synthesis of nano-formulations with consistent size, charge, and surface modifications (such as PEG or targeting peptides) under Good Manufacturing Practice (GMP) conditions is challenging and can be costly. Third, the current literature has a lack of clarity regarding methodologies for optimal dosing, administration routes, and treatment intervals, making it difficult to establish a standardized protocol for Phase I human trials. Future research must address these standardized physicochemical characterizations, optimize GMP-compliant manufacturing, and mandate extended follow-up times for comprehensive biosafety evaluations. This structured approach will be critical for translating NPs into clinically effective therapies for arthritis and other degenerative joint diseases.

### Limitations

This systematic review is subject to certain limitations, notably the inability to conduct meta-analyses due to profound methodological heterogeneity across studies rather than standardized approaches. The studies exhibit immense variation in the models used for arthritis induction (e.g., CFA, CIA, DMM, ACLT, MIA), unstandardized dosing regimens, varying administration routes (intra-articular, oral or intraperitoneal), and inconsistency in control groups. In addition, a limitation of the current literature is the consistently small sample sizes employed across the in vivo studies, which reduces the statistical power and generalizability of the findings. Additionally, bioavailability and toxicity studies were limited in duration (≤ 56 days), highlighting the need to standardize treatment protocols and rigorously evaluate long-term safety. Only a single clinical trial was identified, but it was outside the inclusion criteria. The study is discussed to provide a translational context. However, no eligible clinical data were available for systematic analysis, limiting direct clinical interpretation.

## Conclusions

NP-based therapies might offer a promising approach to managing arthritis by targeting oxidative stress and inflammation, as evidenced by preclinical data from the reviewed studies. Further well-designed preclinical research with standardized protocols, extended follow-up, and comprehensive biosafety evaluation is needed to optimize NP formulations and dosing strategies and advance them into clinical research that will ultimately translate into viable clinical therapies for arthritis and other degenerative joint diseases.

## Supplementary Information

Supplementary Material 1

## Data Availability

Inapplicable.
